# Numerical Simulation Study of Gas-Solid Heat Transfer and Decomposition Processes of Limestone Calcined with Blast Furnace Gas in a Parallel Flow Regenerative Lime Kiln

**DOI:** 10.3390/ma15114024

**Published:** 2022-06-06

**Authors:** Shaopei Duan, Baokuan Li, Wenjie Rong

**Affiliations:** 1 School of Metallurgy, Northeastern University, No. 3-11, Wenhua Road, Heping District, Shenyang 110819, China; duanshaopei_neu@163.com (S.D.); rongwenjie@smm.neu.edu.cn (W.R.); 2 Key Laboratory of Data Analytics and Optimization for Smart Industry, Ministry of Education, Northeastern University, Shenyang 110819, China

**Keywords:** PFR lime kiln, lime calcination, BFG, numerical simulation, PMM, SCM

## Abstract

Quicklime is an essential reducing agent in the steel smelting process and its calcination from limestone is accompanied by considerable energy consumption. As a relatively economical lime kiln, the Parallel Flow Regenerative (PFR) lime kiln is used as the main equipment for the production of quicklime by various steel industries. PFR lime kilns generally use natural gas as the fuel gas. Although natural gas has a high calorific value and is effective in calcination, with the increasing price of natural gas and the pressure saves energy and protect the environment, it makes sense of exploring the use of cleaner energy sources or other sub-products as fuel gas. The use of blast furnace gas (BFG) as a low calorific value fuel gas produced in the steel smelting process has been of interest. This paper therefore develops a set of mathematical models for gas-solid heat transfer and limestone decomposition based on a Porous Medium Model (PMM) and a Shrinking Core Model (SCM) to numerically simulate a PFR lime kiln using BFG in order to investigate the feasibility of calcining limestone with low calorific fuel gas and to provide a valuable reference for future development of such processes and the kiln structure improvement.

## 1. Introduction

The main component of quicklime is calcium oxide (CaO), which is widely used in the metallurgical, construction, agricultural and chemical industries because of its ease of preparation, low price and good reducing properties. Nearly half of the world’s quicklime is used in metal smelting, so the metallurgical industry has the greatest demand for quicklime. Quicklime is obtained by calcining limestone at high temperatures, the main component of which is calcium carbonate (CaCO_3_) with small amounts of silicon dioxide (SiO_2_) and magnesium oxide (MgO).

Among all types of quicklime products, the quantity of quicklime used in metal smelting is not only huge, but there are also strict requirements for its high reducibility. At temperatures less than 1173 K the calcination does not occur and is called “under-burning”; at temperatures above 1423 K the surface porosity becomes smaller and thus less chemically active and is called “hard-burnt” or “over-burnt” At temperatures between 1173 K and 1423 K, calcination results in large surface porosity and strong chemical activity, making it suitable as an excellent reducing agent for metal smelting, which is known as “soft-burnt”.

Many researches have been carried out on the association between calcination and the chemical activity of quicklime.Suju Hao finds the lime activity degree was determined by acid-base titration, and the lime pore distribution was measured by mercury intrusion porosimetry [1]. Vola deal with thermal analyses, burning trials and reactivity tests on 15 carbonate rocks, i.e., pure and impure carbonates, mud-supported and grain-supported limestones, crystalline marbles, and dolomites, used for the production of different lime products in industrial vertical shaft kilns worldwide [2]. Wang does many studies on the relationship between microstructure and physicochemical properties of limestone including, porosity, bulk density, pore size distribution, specific surface area and activity were studied under the condition of rapid heating from room temperature to 1350–1550 °C, the results showed that complete decomposition of limestone (diameter 12.5–15 mm) at 1350 °C, 1450 °C and 1550 °C took 11.7 min, 9.2 min and 6.9 min, respectively [3]. The subject of was to evaluate the influence of lime particle size and lime calcination level as well as slag properties (basicity, viscosity, CaO activity and density) on lime particle dissolution at temperature in the early stage of process blowing time of the basic oxygen furnace (1400 °C) [4].

The Parallel Flow Regenerative (PFR) lime kiln is widely used in the metallurgical industry due to its high daily production capacity (600 t/d–800 t/d), good chemical activity of the product and high thermal efficiency. At the same time, PFR lime kilns are constantly developing and evolving into a variety of improved models.

A three-dimensional steady-state model to predict the flow and heat transfer in a rotary lime kiln is presented [5]. Senegačnik et al. present a case of a remodelled annular shaft kiln, in which the air excess ratio can be reduced to its optimal level by recirculation of recuperator waste gas and its injection into the combustion chambers [6]. The objective of Gutiérrez is to analyze the energy and energy consumption of the calcination process in vertical shaft kilns, in order to identify the factors affecting fuel consumption [7]. The PFR lime kiln has established itself worldwide for the type of product. Piringer explains the functional principle of such lime kilns [8]. Krause present DEM-CFD simulations of the transient processes occurring in an industrial scale PFR-kiln [9].

There are also many CFD numerical simulations for lime kilns. The proposed predictive control method can make the output value of the calcination zone temperature of the lime rotary kiln fast and stable to track the change of the reference value [10]. Suárez builds a regression model for each one of the three prime variables (gas consumption, SO_2_, and NO_x_ emissions) of a lime kiln employed in the paper manufacturing process using the multivariate adaptive regression splines (MARS) method in combination with the artificial bee colony (ABC) technique [11]. Metz evaluates the influence of electric arc furnace dust (EAFD) and lime kiln waste (LKW) [12]. Juneja covers the modeling and control aspects of Lime Kiln process [13]. CFD simulations of an industrial scale rotary kiln for cement clinker production are also conducted [14]. The impact of these coating layers on the clinker production process within a rotary kiln is investigated with CFD simulations [15]. Sagastume and Wenjie analyzed the thermal performance of the lime kiln using the worm-efficiency, pointing out that the performance of a new direction for the analysis of the performance of lime kilns [16,17].

Cheng, et al. [18,19] used the shrinking core model as the basis to find the gas-solid temperature profile inside the ASK by numerical simulation and analyzed the limestone decomposition rate related to the limestone type. Bluhm-Drenhaus [20] and Krause [21] combined the finite volume method (FVM) with the finite element method(FEM) to simulate the temperature field of the gas-solid flow inside the lime kiln and calculated the trajectory of the particles in the kiln with the flow process of the fuel gas. Ning Jingtao [22] studied the calcination of fine limestone using a shrinking core model and confirmed that 1200–1473 K is a more desirable calcination temperature for limestone. The coupled simulation of DEM (discrete element method) and CFD (finite volume method) is a method to solve the stacked bed problem in lime kilns, where the DEM model solves the limestone decomposition behavior while the CFD model simulates the gas-solid two-phase heat exchange and the pressure drop in the stacked bed [23]. Mikulčić, et al. [24] used this coupled approach to study the cement industry for CO_2_ emissions. Meanwhile, a two-energy equation model based on a porous media model is developed by Long Huilong, et al. [25] to simulate the gas-solid chemical reaction in a non-stationary manner, which is in agreement with the experimental results. Shanshan Bu, et al. [26] studied the effect of three particle contact models on the heat transfer in a stacked bed and found that the three particle contact models have a large influence on the calculation results in turbulent flow. Liu, et al. [27] used a gas-burnt lime shaft kiln as a research object and solved the temperature field inside the lime kiln using a local non-thermal equilibrium model.

Previously, in order to ensure the calcination effect, natural gas was generally used as the fuel gas, mixed with air and then burned to generate heat and heat as the heat source for the calcination of limestone. While this traditional process has ensured a good quality of limestone calcination, it has also created significant problems. Firstly, the main component of natural gas is methane, and with the progressively stricter restrictions on greenhouse gas emissions in countries around the world, strict regulations have been made on the relevant equipment for metallurgical enterprises, and PFR lime kilns, as the main equipment consuming natural gas, have to consider energy saving improvements; secondly, the price of natural gas continues to rise due to fluctuations in global political and economic factors, bringing huge cost pressures on the relevant enterprises; finally, in some metallurgical enterprises, blast furnace gas is difficult to be widely used in various production processes because the combustion produces a temperature of only 1673 K to 1773 K, and the utilisation rate is low. In addition, the PFR lime kiln supports 40 mm to 120 mm particle size for calcination when using natural gas as the fuel gas, and there is still a lack of research into the range of particle sizes supported by the change of fuel. In summary, the consideration of low calorific value sub-products, represented by blast furnace gas, as an alternative to natural gas for limestone calcination is gaining interest in the industry. Therefore, this paper makes an attempt in this direction by replacing natural gas with blast furnace gas and establishing a reliable mathematical model of gas-solid heat exchange and calcium carbonate decomposition reaction for the study of its effect, providing a reference basis for future equipment design and the process improvement.

## 2. Mathematical models

The simulation of the PFR lime kiln is based on the Finite Volume Method (FVM), and for any scalar quantity such as temperature, the solution equation as follows:

(1) $$\frac{\partial \rho \phi }{\partial t}+\frac{\partial }{\partial x_{i}}(\rho \vec{u_{i}}\phi -\Gamma \frac{\partial \phi }{\partial x_{i}})=S_{\phi }$$

The mass conservation equation in fluid mechanics theory, also known as the continuity equation, is a fundamental equation of computational fluid dynamics based on a continuous model. It assumes that density, coordinates, time and other variables are continuous in space. Its expression is in the form of Equation (2).

(2) $$\frac{\partial \rho }{\partial t}+\nabla \cdot (\rho \vec{v})=S_{m}$$

Equation (2) is the basic form of the mass conservation equation. The model is used in PFR lime kilns to calculate the mass transfer between gas and solid, for example, the mixing of heating and cooling gases, and the emission of carbon dioxide during decomposition.

The Navier-Stokes equations are equations describing the conservation of momentum in the motion of incompressible fluid, i.e., the sum of the external forces acting on the control body is equal to the rate of change of momentum on the control body with time, and have the following basic form.

(3) $$\frac{\partial (\rho \vec{v})}{\partial t}+\nabla \cdot (\rho \vec{v}\vec{v})=-\nabla p+\nabla \cdot (\overset{=}{\tau })+\rho \vec{g}+\vec{F}$$

In PFR lime kilns, the momentum equation is used to simulate the gas flow and wind resistance variations.

The space inside the lime kiln is filled with limestone particles and the particles move slowly downward, so it can be treated as a moving bed problem inside the lime kiln. The porous media model allows accurate calculation of the pressure drop problem for the moving bed/packed bed, which assumes that the space is occupied by a mixture of solids and fluids.

The introduction of the porous media model in the PFR lime kiln simulation is essentially the introduction of a source term for the momentum loss in the momentum equation to describe the pressure drop (flow wind resistance) generated by the fluid flowing through fluid zone. And the losses originate from the flow viscous resistance and inertial resistance, which is calculated as below.

Ergun equation

(4) $$\frac{\left|\nabla p\right|}{L}=\frac{150\mu {\left(1-\gamma \right)}^{2}}{D_{p}^{2}\gamma^{3}}v_{\infty }+\frac{1.75\rho (1-\gamma )}{D_{p}\gamma^{3}}v_{\infty }^{2}$$

(5) $$\frac{1}{\alpha }=\frac{150{\left(1-\gamma \right)}^{2}}{D_{p}^{2}\gamma^{3}}$$

(6) $$C_{2}=\frac{3.5(1-\gamma )}{D_{p}\gamma^{3}}$$

The shrinking core model assumes that the reactants are spheres, that heat is supplied uniformly from all directions, and that the chemical composition of the raw materials is uniform.

(7) $$\frac{\partial r_{{\mathrm{CaCO}}_{3}}}{\partial t}=-k\cdot \frac{M_{{\mathrm{CaCO}}_{3}}}{\rho_{{\mathrm{CaCO}}_{3}}}\cdot R_{D}$$

(8) $$R_{D}=k_{D}\left(p_{eq}-p_{co_{2}}\right)$$

(9) $$k_{D}=0.0001T_{p}\exp\left(-4026/T_{p}\right)\cdot Y_{T.C}$$

(10) $$p_{\mathrm{eq}}=101325\exp\left[17.74-0.00108T_{i}+0.332\log(T_{i})-22020/T_{i}\right]$$

(11) $$Y_{T.C}=\{\begin{array}{cc} \frac{480}{T_{P}-958} & T_{P}>1150K\\ 2.5 & T_{P}\leq 1150K \end{array}$$

(12) $$\lambda =\frac{4\pi \lambda_{1}\lambda_{2}}{\lambda_{1}\left(\frac{1}{r_{c1}}-\frac{1}{r_{c2.m}}\right)+\lambda_{2}\left(\frac{1}{r_{c1.m}}-\frac{1}{r_{c1}}\right)}$$

Limestone decomposition rate is the volume fraction of decomposed calcium carbonate:

(13) $$X_{S}=1-\frac{r_{c2}^{3}}{r_{c1}^{3}}$$

The temperature equations for gas-solid convective heat transfer, internal and external particle heat conduction, and between solid particles

(14) $$\frac{\partial }{\partial t}\left[\varphi_{{\mathrm{CaCO}}_{3}}\rho_{i}c_{pi}T_{i}\right]+\nabla \left({\vec{v}}_{\mathrm{down}}\varphi_{{\mathrm{CaCO}}_{3}}\rho_{i}c_{pi}T_{i}\right)=\lambda \left(1-\gamma \right)\left(T_{o}-T_{i}\right)/V_{s}-k\cdot Q_{D}\Delta H_{R}$$

(15) $$Q_{D}=(1-\gamma )\frac{4\pi r_{c1}^{2}}{V_{p}}\times R_{D}$$

Energy equation of the external particle (calcium oxide)

(16) $$\begin{array}{l} \frac{\partial }{\partial t}(\varphi_{\mathrm{CaO}}\rho_{o}c_{po}T_{o})+\nabla \cdot ({\vec{v}}_{down}\varphi_{\mathrm{CaO}}\rho_{o}c_{po}T_{o})=\\ \nabla \cdot \left(\left(k_{\mathrm{CaO}}+e_{b}\right)\nabla T_{o}\right)\;+a_{v}h_{v}\left(T_{g}-T_{o}\right)-\lambda \left(1-\gamma \right)\left(T_{o}-T_{i}\right)/V_{s} \end{array}$$

The radiation equivalent thermal conductivity treatment of porous media used for radiation between solids [28]

(17) $$e_{b}=16\sigma T_{o}^{3}/\left(3\beta \right)$$

The convective heat transfer coefficient $h_{v}$ is approximately calculated by flow-across tube bundle model in the heat exchanger model [29], which is calculated as following equations

(18) $$h_{v}=\frac{Nu\cdot \lambda_{g}}{l_{z}}$$

(19) $$Nu=Pr_{}^{1/3}\cdot \frac{1.6274Re_{}^{-0.575}}{\gamma }$$

(20) $$l_{z}=0.0178\rho^{0.596}$$

(21) $$Re=\frac{\rho_{g}\cdot D_{p}\cdot u}{\mu }$$

(22) Pr=ν/a

(23) $$a=\frac{\lambda_{g}}{\rho_{g}c_{pg}}$$

(24) $$a_{v}=(1-\gamma )\times \frac{S_{p}}{V_{p}}$$

The gas energy equation

(25) $$\frac{\partial }{\partial t}\left(\gamma \rho_{g}c_{pg}T_{g}\right)+\nabla \left(\gamma \vec{v}\rho_{g}c_{pg}T_{g}\right)=\nabla \cdot \left(k_{g}\nabla T_{g}\right)+a_{v}h_{v}\left(T_{o}-T_{g}\right)$$

(26) $$\varepsilon =\left(1-\frac{M_{\mathrm{CaO}}/\rho_{CaO}}{M_{CaCO_{3}}/\rho_{CaCO_{3}}}\right)\times X_{S}$$

(27) $$\varphi_{g}=(1-\gamma )\cdot \varepsilon +\gamma$$

(28) $$\varphi_{CaCO_{3}}=(1-\gamma )\times (1-X_{S})$$

(29) $$\varphi_{CaO}=1-\varphi_{g}-\varphi_{CaCO_{3}}$$

## 3. Physical Models

### 3.1. Geometric Model

**Figure 1:**
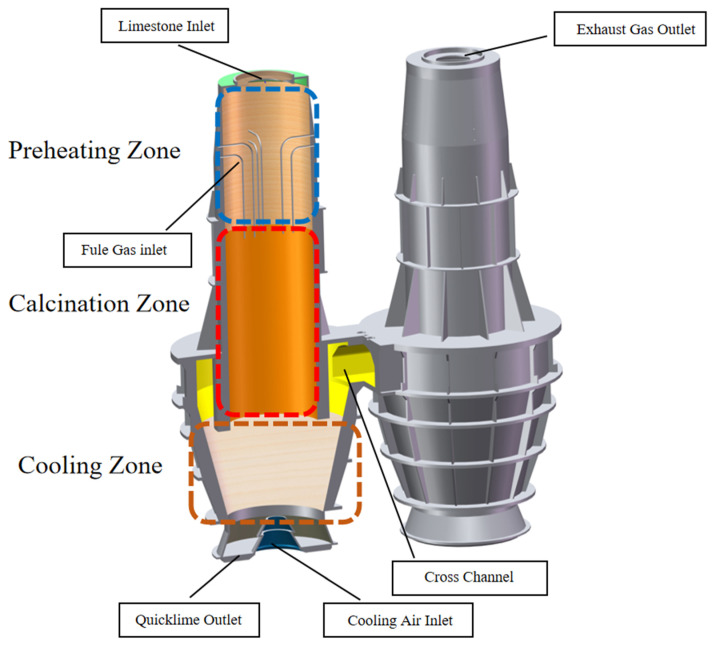
Schematic diagram of the PFR lime kiln components.

The PFR lime kiln has a total height of 20 m and is divided into two chambers, which is connected by a cross channel. Each chamber is divided into a preheating zone, a calcination zone and a cooling zone from top to bottom. The preheating zone is 6 m high, The upper part is the limestone inlet and also the exhaust gas outlet, and the lower part has 8 fuel gas jets. The height of the calcination zone is 9 m. Below the calcination zone is the cooling zone, which is 5 m high and is surrounded by a channel in the upper outer ring, which gradually contracts downwards in the middle to the plane of the cooling air inlet and then gradually expands and extends to the CaO outlet (Figure 1). Chamber 2 has the same components as chamber 1.

A structural grid is used to discretize the entire computational domain. In the region of structurally complex calcination and preheating zones, the grid is sufficiently refined. The grid resolution affects not only the computational time and resources, but also the reliability of the simulation results. The sensitivity for grid numbers of 4.6 million, 5.2 million and 5.5 million was examined in this PFR lime kiln model to obtain grid independent results. The comparison results show that a denser grid has a minor effect on the computational results.

### 3.2. Operating Procedures

**Figure 2:**
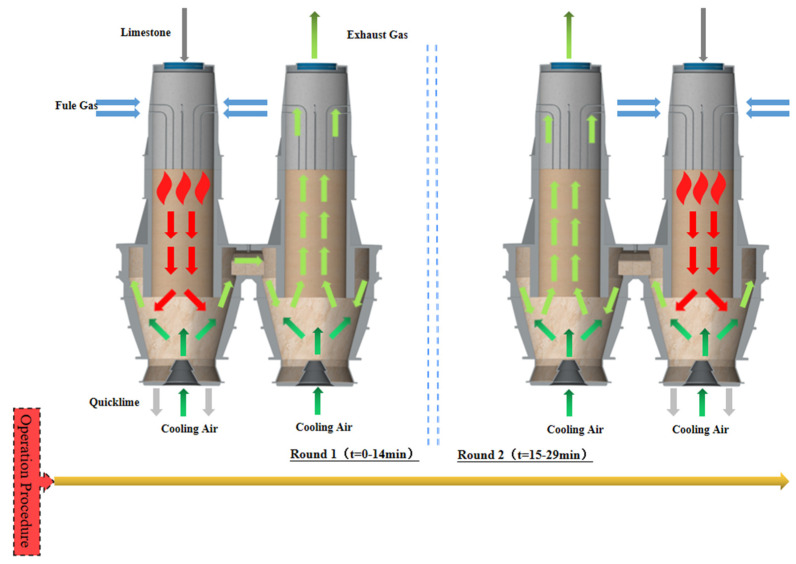
Schematic diagram of the operating procedure of the PFR lime kiln.

The PFR lime kiln consists of two chambers, with the fuel gas in Chamber 1 injected into the calcination zone to calcine the limestone while the fuel gas jet in Chamber 2 does not operate. The main component of limestone is the calcium carbonate, which is heated at high temperatures to decompose into calcium oxide and carbon dioxide, a process known as limestone calcination. When calcination begins, the high temperature gas heats the calcium carbonate, which starts to decompose into calcium oxide and carbon dioxide from the surface to the inside, and as the decomposition process proceeds, the material gradually moves down and from the calcination zone into the cooling zone. Cooling air is blown into the cooling zone from the bottom up by the cooling air inlet to quickly cool the material that has just entered the cooling zone so that its chemical activity is not reduced by the high temperature. The material is cooled as it gradually moves down and is eventually discharged through the CaO outlet. The high temperature gas is involved in the calcination and the temperature is reduced, while the cooling air is involved in the cooling and the temperature is increased. The two are mixed in the cross channel to form the exhaust gas. The heat carried by the exhaust gas has a preheating effect on the unreacted material in Chamber 2, while the cooling air is continuously blown into the Chamber 2 cooling gas inlet to enhance the exhaust gas convection and accelerate the efficiency of the exhaust gas upwards. Finally, after passing through the material in the calcining and preheating zones in Chamber 2, the exhaust gas is discharged by the upper exhaust gas outlet, while the limestone inlet in Chamber 1 is only feeding the material at this time and is not operating as an exhaust gas outlet. After 15 min of operation of Chamber 1, fuel gas is instead injected by the fuel gas jet in Chamber 2, while Chamber 1 functions as an exhaust gas outlet for 15 min. The PFR lime kiln completes one cycle every 30 min and continues to operate (Figure 2).

### 3.3. Boundary Conditions and Calculation Methods

As the outer walls of the PFR lime kiln are well insulated, the inner surface of the kiln is used as an adiabatic boundary. The interior of the kiln is divided into two fluid domains, the porous media area filled with the calcium carbonate and the gas area where only the exhaust gas is present. The boundary below the fuel gas nozzle is used as the fuel gas inlet boundary, while the rest of the nozzle is used as the exterior. Also, the temperature of the blast furnace gas after combustion is used as the fuel gas inlet temperature and other calculation conditions are shown in Table 1.

Density, thermal conductivity and void fraction is determined in the laboratory.

The semi-implicit method of coupled pressure equations (referred to as the SIMPLE algorithm) was used to solve the coupled velocity and the pressure problem in the process of solving the equations; two equations in the turbulence equations were solved in the first-order windward difference format, and the remaining equations were treated by the second-order windward difference; the convergence factor of each equation was set to 10^−6^.

The following hypotheses will also be established in this study in order to exclude any interference to the results.

i. The limestone is composed exclusively of calcium carbonate, disregarding the influence of impurities that are in fact present in the limestone, such as MgO, on the calcination results.
ii. The limestone raw material is spherical and the particle size refers to the diameter.
iii. Ignoring the effect of airflow on the movement of the material.

## 4. Results and Analysis

**Figure 3:**
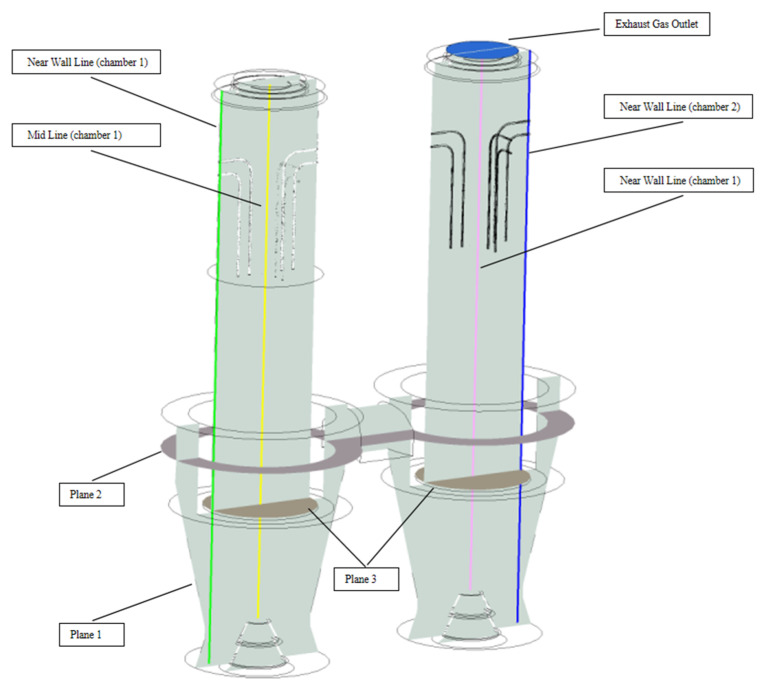
Schematic diagram of the analysis position.

A number of locations within the PFR lime kiln were taken for analysis, as shown in Figure 3, where the two intermediate lines are located in the geometric centre of the chambers called mid lines and the near wall lines are located 40 mm from the chamber side wall, with at least one material particle accommodated between the line and the side wall, which is not only farther away from the fuel gas inlet and poorly heated, but is also affected by the side wall effect, with differences in particle movement and decomposition downwards from mid lines. The exhaust gas temperature was analysed by taking the exhaust gas outlet above chamber 2. The central profile Plane 1 is taken to show the overall distribution of all fields. Plane 2 is taken in the centre of the intermediate channel to analyse the mixture of exhaust gas and cooling air after calcination. Plane 3 is taken at the intersection of the calcining and cooling zones and analyses the surface temperature of the solids after calcination, the core temperature and the decomposition of the limestone.

### 4.1. Gas Velocity Field

**Figure 4:**
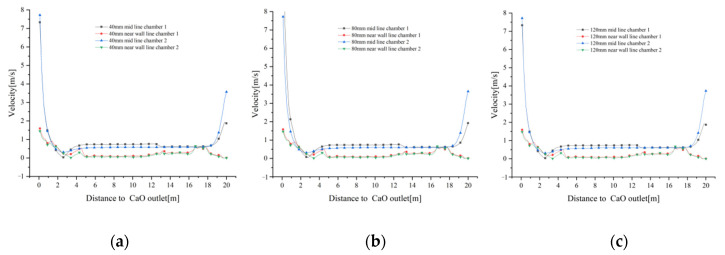
Gas velocity variation on the mid lines and near wall lines for 40 mm, 80 mm and 120 mm particle sizes. (**a**) Gas velocity variation at 40 mm; (**b**) Gas velocity variation at 80 mm; (**c**) Gas velocity variation at 120 mm.

The gas velocity distributions on the mid lines and near wall lines at 40 mm, 80 mm and 120 mm particle sizes are shown in Figure 4. At a distance of 14 m from the CaO outlet, fuel gas enters the calcination zone from the boundary to heat the limestone, and as the distance gradually decreases, the mid lines gas velocity increases, but is blocked by the solid material and the increase is small. Until about 5 m from the CaO outlet, the gas velocity decreases and then sharply increase to about 7 m/s at about 2 m from the CaO outlet due to the effect of cooling air blowing in. Due to the high temperature and low density of the fuel gas, some of it rises into the preheating zone after being blown into the calcination zone of the lime kiln. In addition, after the cooling air has cooled the high temperature material, some of the exhaust gas does not flow through the intermediate passage towards chamber 2, but enters the preheating zone via the calcination zone of chamber 1.

The gas velocity distribution of the near wall line in the calcination zone shows an opposite trend to that of the mid line. As one approaches the CaO outlet, the near wall line gas velocity decreases and the gas reaches the cooling zone with a velocity close to that of mid lines gas velocity. However, at closer distances from the CaO outlet, the near wall line gas velocity does not show a sharp increase in velocity similar to mid line gas velocity, but increases slowly to below 2 m/s. Similarly, in the preheating zone, the near wall line gas velocity is less affected by the rising high temperature fuel gas and cooling air convergence, and the gas velocity is smaller, approaching 0 near the top limestone inlet. It should be noted that the velocity at 14 m from the CaO outlet is not the fuel gas inlet velocity, as neither mid line nor the near wall line passes through the fuel gas inlet boundary. (Figure 4a–c)

**Figure 5:**
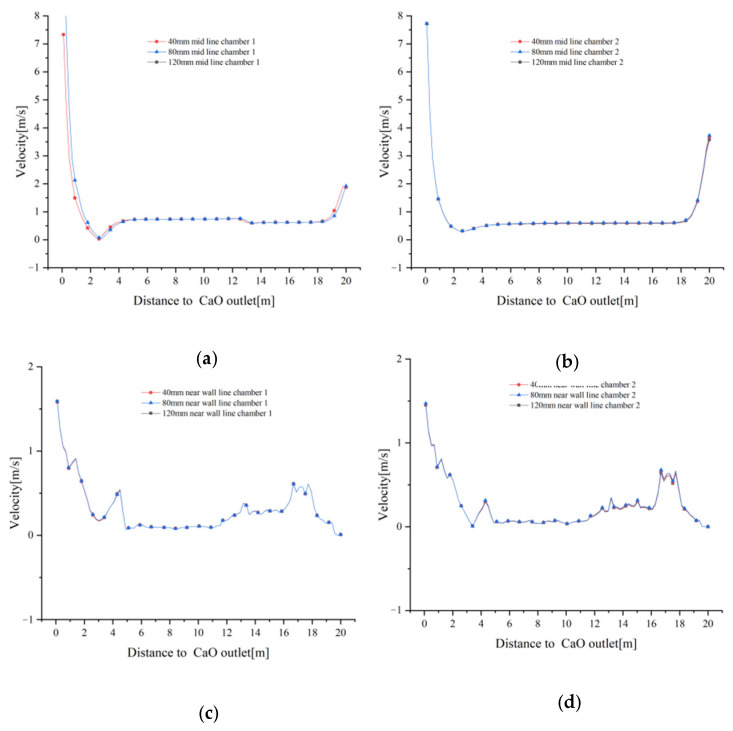
Comparison of gas velocities at mid lines and near wall lines in chamber 1 and 2. (**a**) Change in gas velocity at the mid line of chamber 1; (**b**) Change in gas velocity at the mid line of chamber 2; (**c**) Change in gas velocity at the near wall line of chamber 1; (**d**) Change in gas velocity at the near wall line of chamber 2.

Similarly, the variation of gas velocity tends to be the same for the three particle sizes of 40 mm, 80 mm and 120 mm (Figure 5a–d), indicating that the effect of different void fractions on gas velocity is small.

Combined Figure 4 and Figure 5 show that the gas velocity varies less with particle size. Whereas the velocity variations in the centre of the chamber (mid lines) and the kiln side wall (near wall lines) converge in the calcination zone, they show different variations in the preheating and cooling zones. The centre of the cooling air inlet is on the same axis as the centre of the chamber, which inevitably leads to higher velocities in the centre of the chamber. The side walls of the chamber, on the other hand, are less affected by the fuel gas and cooling air, and their velocity variation over the sections is also less.

### 4.2. Gas Temperature Field

**Figure 6:**
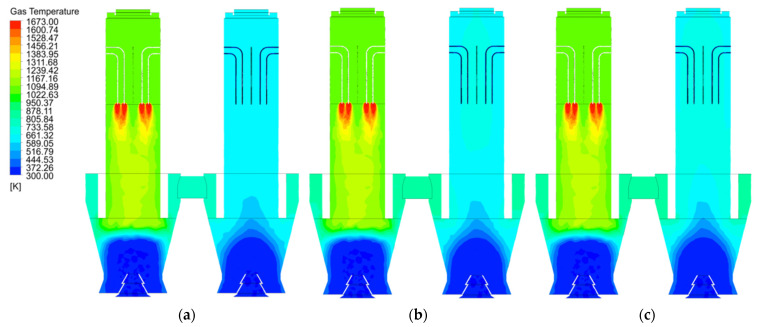
Gas temperature distribution at 40 mm, 80 mm and 120 mm particle sizes. (**a**) Gas temperature distribution at 40 mm (**b**) Gas temperature distribution at 80 mm (**c**) Gas temperature distribution at 120 mm.

As the gas temperature distribution is directly influenced by the gas velocity distribution, there is little difference in the gas temperature distribution at 40 mm, 80 mm and 120 mm particle sizes (Figure 6a–c). At 40 mm particle size, the temperature distribution in the calcination zone of chamber 2 (where calcination does not occur) is uniform (Figure 6a), while at 80 mm particle size, the temperature in the centre of the calcination zone of chamber 2 is significantly higher than that in the side walls (Figure 6b), and as the particle size increases to 120 mm, the temperature in the centre of both the calcination and preheating zones of chamber 2 is higher, with less difference between the temperature in the near wall zone and the center (Figure 6c).

**Figure 7:**
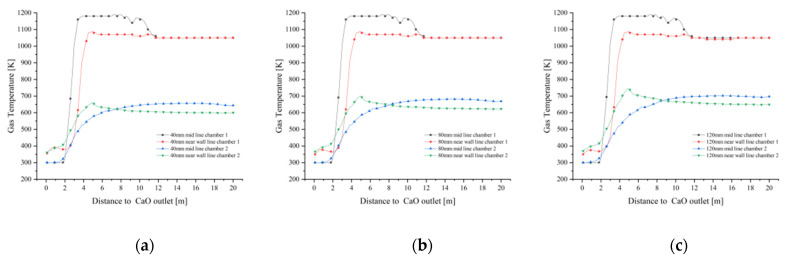
Gas temperature variation on the mid and near wall lines for 40 mm, 80 mm and 120 mm particle sizes. (**a**) Gas temperature variation at 40 mm (**b**) Gas temperature variation at 80 mm (**c**) Gas temperature variation at 120 mm.

As shown in Figure 7a, the temperature of the near wall line of chamber 1 at 40 mm particle size varies little from the preheating zone to the lower part of the calcination zone, until the junction of the cooling zone, where the gas temperature is influenced by the cooling air and decreases to around 350 K. The temperature of the mid line of chamber 1 remains similar to the near wall line in the preheating zone, but increases rapidly up to 1200 K upon entering the calcination zone. The mid line of chamber 1 remains at a similar temperature to the near wall line in the preheating zone, however, the temperature increases rapidly in the calcination zone due to the high temperature of the fuel gas up to 1200 K. After entering the cooling zone, the mid line gas temperature is also affected by the cooling air and decreases rapidly to around 350 K. The gas temperature at the mid line of chamber 2 varies more gently with the height of the chamber and only changes significantly between the cooling zone and the cross channel. The change in gas temperature in chamber 1 at 80 mm and 120 mm particle size is more or less the same as the change in gas temperature at 40 mm (Figure 7b,c).

**Figure 8:**
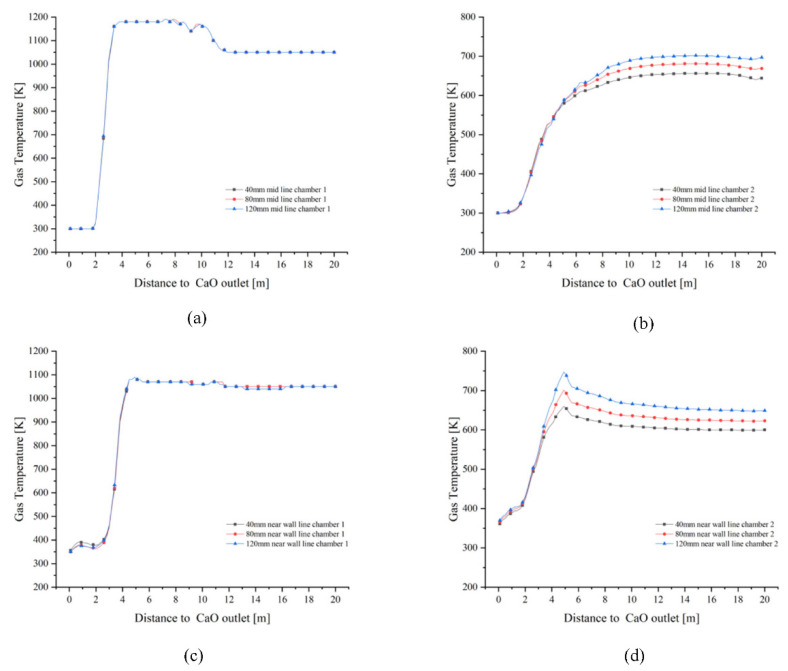
Comparison of gas temperature variations in the mid line and near wall line of chamber 1 and 2. (**a**) Change in gas temperature at the mid line of chamber 1; (**b**) Change in gas temperature at the mid line of chamber 2; (**c**) Change in gas temperature at the near wall line of chamber 1; (**d**) Change in gas temperature at the near wall line of chamber 2.

The variation in mid line and near wall line temperatures in chamber 1 is essentially the same for all three particle sizes (Figure 8a,c). In chamber 2, the variation in mid line and near wall line gas temperatures is more pronounced with lime kiln height for all three particle sizes. 120 mm particle size chamber 2 mid line gas temperatures is higher than 80 mm and 40 mm chamber 2 mid line gas temperatures (Figure 8b). Similarly, the near wall gas temperature of chamber 2 for 120 mm particle size is higher than the near wall gas temperature of chambers 2 for 80 mm and 40 mm (Figure 8d).

**Figure 9:**
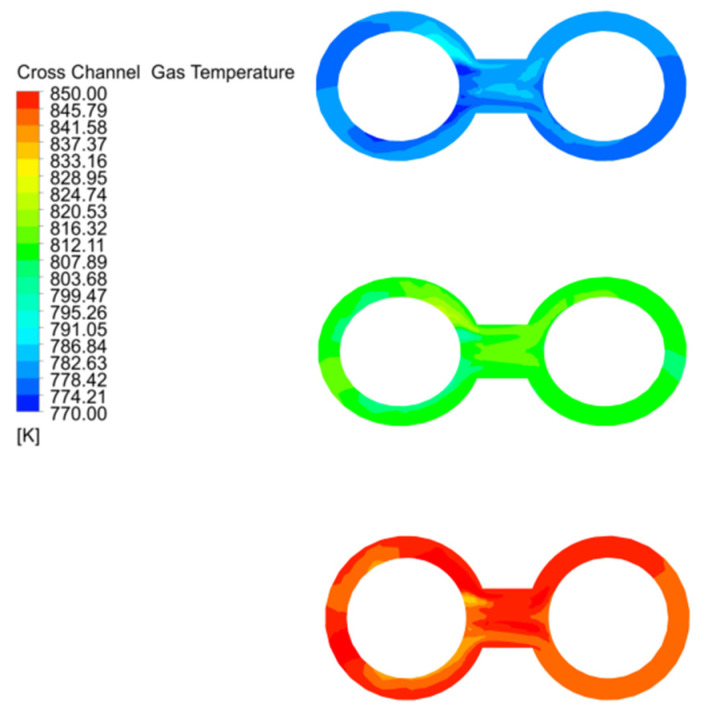
Gas temperature distribution and variation in the cross channel at 40 mm, 80 mm and 120 mm particle sizes.

The cross channel has only high temperature exhaust gas and no solid material, so the temperature variation is more pronounced than in the chambers, and is characterised by uneven gas mixing and temperature distribution in the outer ring of chamber 1, while entering the centre of the channel, the mixing is uniform and the average temperature is highest at this point, and gradually decreases as the gas enters the outer ring of chamber 2 (Figure 9).

**Figure 10:**
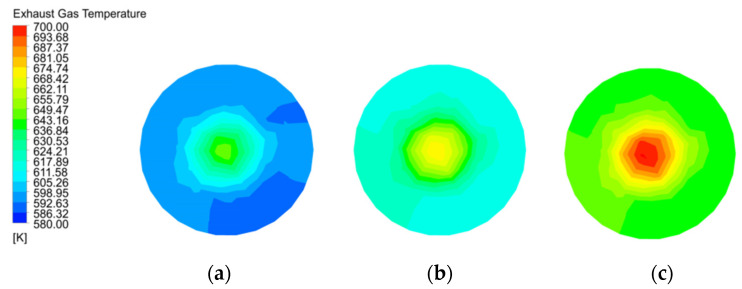
Gas temperature distribution and variation in exhaust gas outlet at 40 mm, 80 mm and 120 mm particle sizes. (**a**) Gas temperature in exhaust gas outlet of 40 mm; (**b**) Gas temperature in exhaust gas outlet of 80 mm; (**c**) Gas temperature in exhaust gas outlet of 120 mm.

The gas temperature distribution at the exhaust gas outlet in Figure 10 matches the gas temperature in Figure 6. 120 mm particle size has the highest gas temperature at the exhaust gas outlet, followed by 80 mm, while 40 mm particle size has the lowest gas temperature. The gas temperature at the centre of the exhaust gas outlet is also higher than near wall, which is consistent with the results in Figure 7.

Combined with the gas temperature variations and distributions in Figure 6, Figure 7, Figure 8, Figure 9 and Figure 10, the gas temperature distributions in chamber 1 are similar for 40 mm, 80 mm and 120 mm particle sizes, while the gas temperature in chamber 2 increases as the particle size gradually increases, as reflected in the cross channel and the exhaust gas outlet cross section. This indicates that the gas temperature distribution varies with the particle size (void fraction) for a given gas velocity. The effect of particle size (void fraction) on gas temperature decreases as the gas velocity increases.

### 4.3. Solid Surface Temperature

For CaO used in reduction reactions in metallurgical processes, the chemical activity is the most important indicator, and maintaining a solid surface temperature of no more than 1423 K during calcination is a decisive parameter in maintaining chemical activity. If the solid surface pore size is reduced over a long period of time above 1423 K, the chemical activity decreases and is referred to as “hard-burnt” or “over-burnt”, so it is important to maintain a reasonable solid surface temperature.

**Figure 11:**
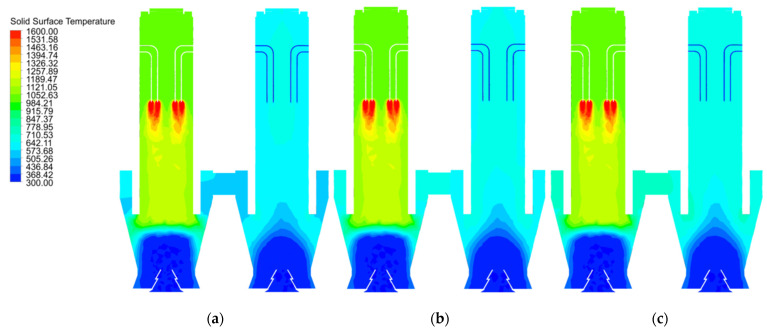
Solid surface temperature distribution at 40 mm, 80 mm and 120 mm particle sizes. (**a**) 40 mm solid surface temperature distribution (**b**) 80 mm solid surface temperature distribution (**c**) 120 mm solid surface temperature distribution.

The 40 mm solid material briefly reaches about 1600 K near the fuel gas inlet in the calcination zone of chamber 1, then the temperature drops rapidly as the material moves down into the cooling zone, where the solid surface temperature is rapidly cooled to room temperature by the cooling air. In contrast, the solid surface temperature in the preheating zone does not exceed 1200 K (Figure 11a). The solid material surface temperatures at 80 mm and 120 mm particle size also show similar temperature distributions, but in chamber 2 the solid material surface temperature distribution at 80 mm and 120 mm particle size differs from that at 40 mm, which is more in line with the gas temperature distribution in Figure 6.

**Figure 12:**
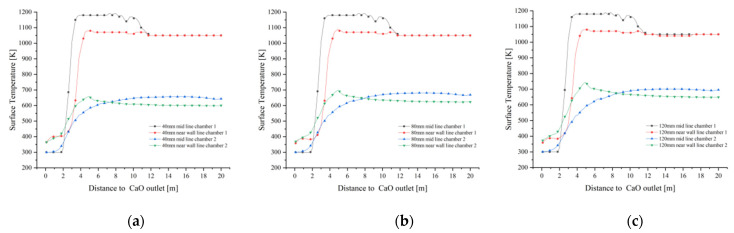
Variation of solid surface temperature on the mid lines and near wall lines for 40 mm, 80 mm and 120 mm particle sizes. (**a**) 40 mm solid surface temperature variation (**b**) 80 mm solid surface temperature variation (**c**) 120 mm solid surface temperature variation.

As shown in Figure 12a–c, the solid surface temperatures at the mid and near wall lines of chamber 1 are similar in the preheating zone and gradually increase as they move from the calcination zone towards the CaO outlet, with a greater temperature gradient at the mid line than at the near wall line. The mid line and near wall line solid surface temperatures are rapidly cooled by the cooling air until they reach the cooling zone, where the temperature rapidly decreases to around 300 K to 350 K. Chamber 1 shows similar variations for all three particle sizes: 40 mm, 80 mm and 120 mm. In chamber 2, however, the temperature of the near wall line is characterised by a larger particle size and a higher surface temperature due to the influence of the high temperature exhaust gas brought in by the cross channel at different temperatures.

**Figure 13:**
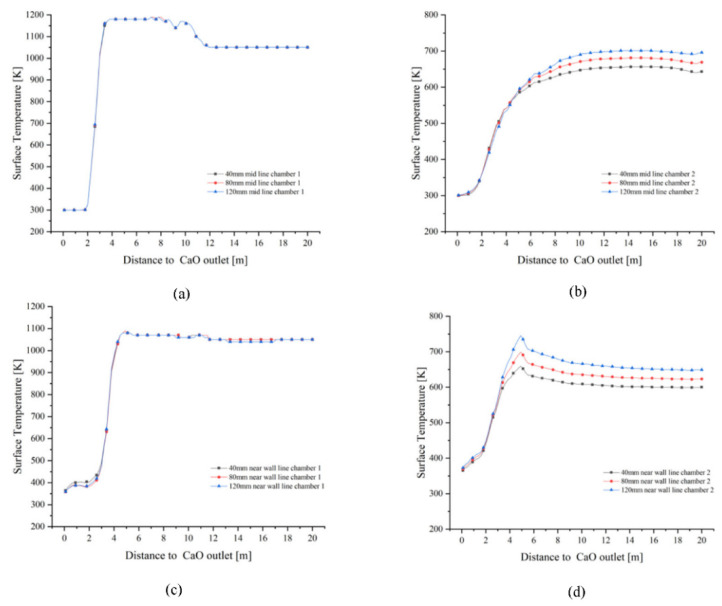
Comparison of solid surface temperature variations in the mid line and near wall line of chamber 1 and 2. (**a**) Change in solid surface temperature at the mid line of chamber 1; (**b**) Change in solid surface temperature at the mid line of chamber 2; (**c**) Change in solid surface temperature at near wall line of chamber 1; (**d**) Change in solid surface temperature at near wall line of chamber 2.

The mid line and near wall line solid surface temperatures of chamber 1 converge for each particle size (Figure 13a,c). In the preheating zone, the solid surface temperature remains around 1050 K. After calcinate in the calcining zone, the mid line solid surface temperature rises close to 1200 K and the near wall line solid surface temperature reaches 1100 K. Neither exceeds 1423 K, which does not lead to hard-burnt and reduced chemical activity. The trend in the mid line and near wall line solid temperatures in chamber 2 correlates with the change in gas temperature, with the largest solid surface temperature gradient at 120 mm particle size reaching 700 K in the mid line and 750 K in the near wall line, and up to 650 K in the mid line and near wall line line at 40 mm.

**Figure 14:**
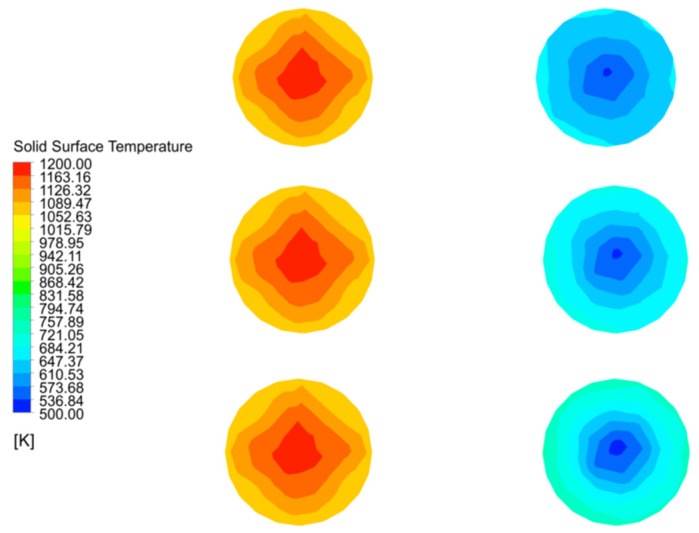
Distribution and variation of solid surface temperatures in double chambers on Plane 3.

Plane 3 is the interface between the calcination and cooling zones, as shown in Figure 14 which reflects the temperature field of the solid material for 40 mm, 80 mm and 120 mm particle sizes before entering the cooling zone. At particle sizes 40 mm and 80 mm, the solid surface temperature distribution in chamber 1 is basically the same, with a maximum value of 1170 K, while the 80 mm particle size has a higher solid surface temperature gradient in chamber 2 than at 40 mm particle size, with a maximum temperature of 690 K, while the 40 mm particle size is only about 650 K. The maximum temperature in chamber 1 is still around 1170 K when the particle size is 120 mm, but the distribution is wider than at 40 mm and 80 mm, and the maximum solid surface temperature in chamber 2 reaches 740 K. In addition, the lowest surface temperature for all three particle sizes in chamber 2 is around 510 K.

The solid surface temperature did not reach the upper limit of 1423 K hard-burnt temperature, which is also related to the fact that the heat of combustion of BFG is lower than the heat of combustion of natural gas. In addition, the near wall line has a significant difference between the downward velocity and the temperature gradient of the material and the mid line material due to the side wall effect. Although the gas temperature fields converge for all three particle sizes, the solid surface temperature still shows a greater temperature gradient at 120 mm in chamber 2 as it gradually approaches the cooling zone. In contrast, the solid surface temperature in chamber 1 does not change significantly with particle size.

### 4.4. Solid Core Temperature

During the calcination of limestone, the temperature must be greater than 1173 K before decomposition reactions begin. As the decomposition reaction progresses from the outside to the inside, the core temperature rises at a slower rate and eventually there may be a part of the core with a temperature less than 1173 K where no decomposition reaction takes place, a situation generally referred to as “under burning”. The “under burning” means that the limestone has not decomposed completely.

**Figure 15:**
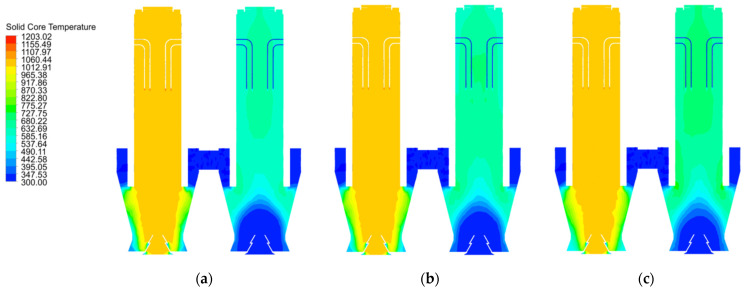
Solid surface temperature distribution at 40 mm, 80 mm and 120 mm particle sizes. (**a**) 40 mm solid core temperature distribution (**b**) 80 mm solid core temperature distribution (**c**) 120 mm solid core temperature distribution.

As shown in Figure 15a, the core temperatures in both the calcination and preheating zones at 40 mm particle size reach above 1000 K, while the core temperature in the central part of the cooling zone reaches above 1000 K. The core temperature near wall drops rapidly. On the one hand, this is due to the fact that the combustion heat of the fuel gas cannot reach a long distance, and on the other hand, it is also due to the fact that the cooling air cools the solid in the cooling zone so quickly that not only the surface temperature of the material drops, but also the core temperature of part of the material. The core temperature in the cooling zone at 120 mm particle size is significantly smaller than that at 1000 K (Figure 15c). The core temperature distribution in chamber 2 for the three particle sizes increases with increasing particle size, resulting in a more pronounced increase in the area with higher core temperatures.

**Figure 16:**
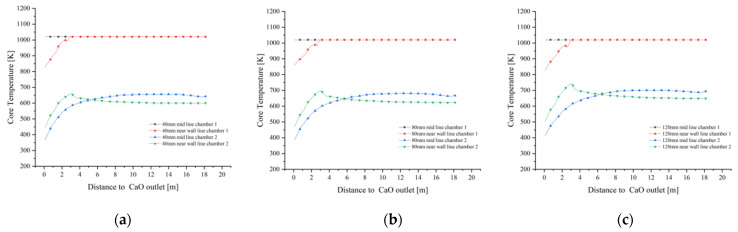
Solid core temperature variation on the mid line and near wall line for 40 mm, 80 mm and 120 mm particle sizes. (**a**) Variation in solid core temperature at 40 mm (**b**) Variation in solid core temperature at 80 mm (**c**) Variation in solid core temperature at 120 mm.

In combination with Figure 15a,b and Figure 16a,b, the variation of the mid line and near wall line temperatures in chamber 1 are essentially the same for 40 mm and 80 mm grain sizes, while for 120 mm grain size, the mid line solid core temperature remains the same as the former, while the near wall line shows a greater drop as it enters the cooling zone (as in Figure 16c). For the solid core temperature in chamber 2, the changes in the mid line and near wall line are essentially the same, remaining at around 650 K and 550 K respectively.

**Figure 17:**
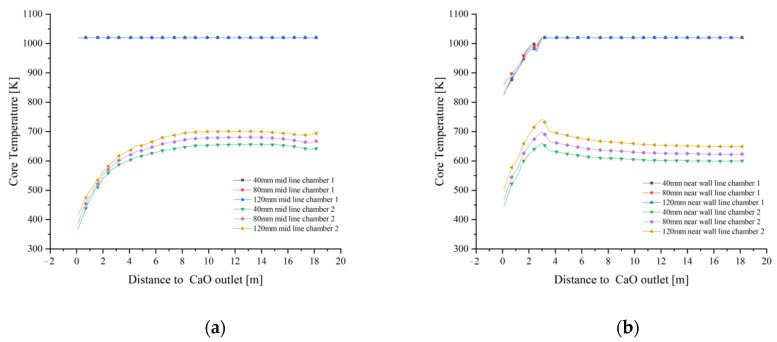
Comparison of mid and near wall line solid core temperature variations. (**a**) Variation in solid core temperature at the mid line (**b**) Variation in solid core temperature at the near wall line.

As shown in Figure 17a,b, the core temperatures in the mid line of chamber 1 are essentially the same, with minor differences in the near wall line only in the cooling zone. In contrast, the core temperatures for the 40 mm, 80 mm and 120 mm particle sizes at the mid line and near wall lines of chamber 2 show significant differences. During the upward movement away from the cooling zone of chamber 2, the solid core temperature reaches 700 K at the mid line of the 120 mm grain size and even 750 K at the near wall line at one point and stabilises around 650 K during the upward movement, while the temperature gradient decreases at the mid line and near wall line of the 80 mm and 40 mm grain sizes, with the maximum temperature gradient reaching over 50 K.

**Figure 18:**
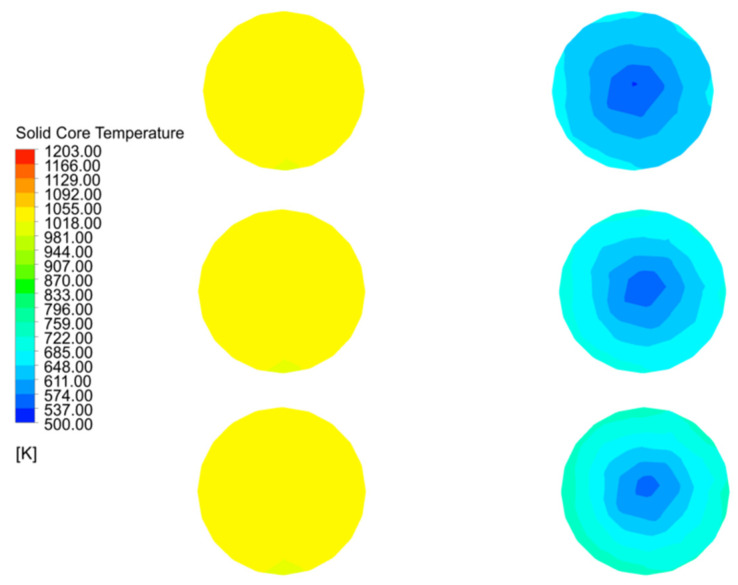
Solid core temperature distribution on double chambers and solid core temperature variation in chamber 2 on Plane 3.

Combining the temperature variations of chamber 1 in Figure 17a,b, it can be seen that the core temperature variations of Plane 3 at the interface between the calcination and cooling zones are mainly manifested in chamber 2, therefore the core temperature analysis of Plane 3 is also for chamber 2, as shown in Figure 18.

The maximum temperature of Plane 3 at the interface of the calcination and cooling zones in chamber 2 at 40 mm particle size is 650 K and the minimum temperature is 550 K. As the particle size increases to 80 mm, the core temperature reaches 740 K and 560 K respectively, and as the particle size continues to increase to 120 mm, the maximum core temperature basically remains unchanged at 740 K, but the minimum temperature rises to around 575 K. There is a significant reduction in the temperature difference.

The core temperature distribution and changes in the solids at 40 mm, 80 mm and 120 mm particle sizes shows that the core temperature in chamber 1 varies little with particle size, especially at 40 mm and 80 mm, and does not change much until 120 mm. The main reason for this is that as the decomposition reaction continues, the core temperature rises more slowly in the larger particle sizes, and by the time it reaches the cooling zone, the core of some of larger solids has not yet risen to a sufficient temperature and is affected by the cooling air and no longer continues to rise. The core temperature contrast in chamber 2 is even more pronounced, i.e., the temperature gradient is smaller for the smaller particle sizes and larger for the larger particle sizes, mainly because the smaller particle sizes warm up faster and the heat is more evenly distributed, while the larger particle sizes show an external hot and internal cold distribution of core temperature at the same time.

### 4.5. Limestone Decomposition Field

**Figure 19:**
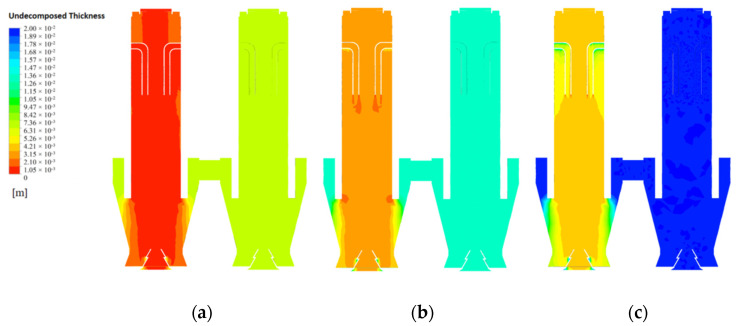
Distribution of undecomposed thickness of CaCO_3_ at 40 mm, 80 mm and 120 mm particle sizes. (**a**) undecomposed thickness of CaCO_3_ at 40 mm (**b**) undecomposed thickness of CaCO_3_ at 80 mm (**c**) undecomposed thickness of CaCO_3_ at 120 mm.

The calcination of limestone is completely dependent on the remaining calcium carbonate thickness and therefore the limestone decomposition is represented by the distribution of undecomposed thickness (e.g., Figure 19a–c). As the particle size gradually increases, the decomposition reaction becomes more difficult to carry out and the remaining limestone thickness no more decreases, indicating that the limestone reaction gradually increases in unreacted thickness as the particle size increases.

**Figure 20:**
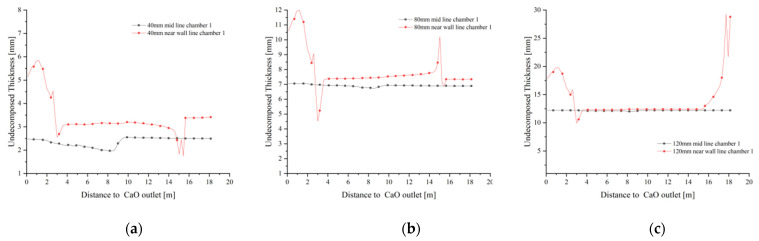
Variation of undecomposed thickness of CaCO_3_ at 40 mm, 80 mm and 120 mm particle sizes. (**a**) Variation of undecomposed thickness of CaCO_3_ at 40 mm (**b**) Variation of undecomposed thickness of CaCO_3_ at 80 mm (**c**) Variation of undecomposed thickness of CaCO_3_ at 120 mm.

At 40 mm particle size, the undecomposed thickness of the chamber 1 calcination zone gradually decreases as the mid line moves down towards the CaO outlet, and the undecomposed thickness remains the same in the upper part of the preheating and calcination zones, remaining at around 2.5 mm after reaching the cooling zone. The undecomposed thickness near the wall line is above 3.5 mm in the preheating zone until it rapidly decreases and remains around 3 mm after entering the calcining zone. A similar trend was observed for the near wall line at 80 mm particle size, where the undecomposed thickness was close to 12 mm, while the undecomposed thickness of the mid line remained around 7 mm and did not change significantly with height (Figure 20b). At 120 mm grain size, the mid line undecomposed thickness is the same as at 80 mm, while the near wall line shows a large difference. The undecomposed thickness of the near wall line was as high as 30 mm in the preheating zone, and only after entering the calcination zone did the undecomposed thickness decrease significantly and was similar to the decomposition of the 40 mm and 80 mm grain sizes (Figure 20c).

**Figure 21:**
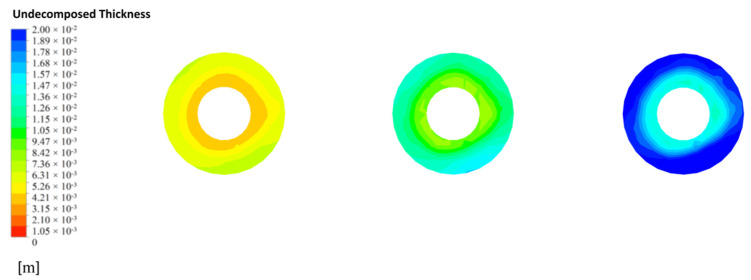
Variation of 40 mm, 80 mm and 120 mm undecomposed thickness of CaO outlet.

At the CaO outlet, the undecomposed thickness distribution is shown in Figure 21. At a particle size of 40 mm, the undecomposed thickness near the inner side is small, with an average undecomposed thickness of about 3 mm, and increases to 7 mm as it moves towards the outer side, while at a particle size of 80 mm, the undecomposed thickness reaches 7 mm on the inner side and more than 13 mm on the outer side. The decomposition distribution at all grain sizes indicates that the side wall effect has a significant influence on the final decomposition, especially at larger grain sizes where the side wall effect has a greater influence on the decomposition process.

**Figure 22:**
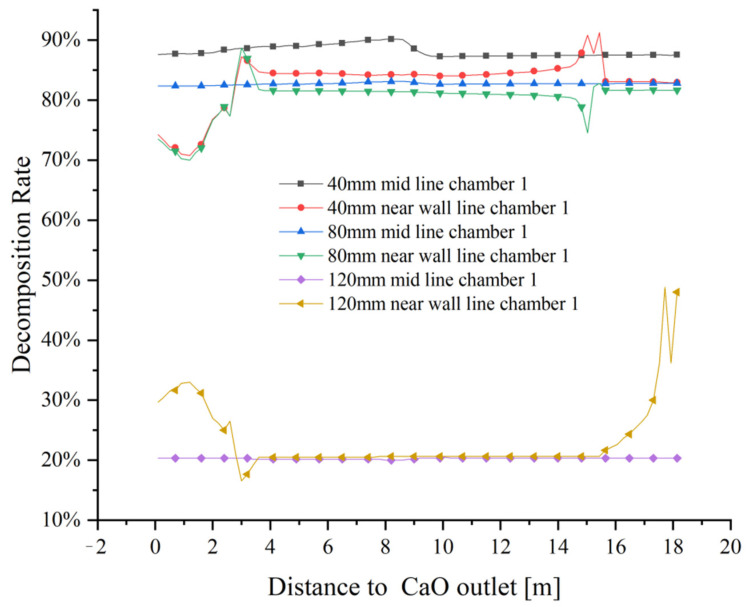
Variation in mid lines and near wall lines decomposition rates at 40 mm, 80 mm and 120 mm particle sizes.

The variation of limestone decomposition rates for the three sizes is shown in Figure 22. At 40 mm particle size, the mid line limestone decomposition rate is close to 90%, while the near wall decomposition rate is between 75% and 85%. The mid line decomposition rate reaches about 83% with 80 mm particle size, while the near wall decomposition rate is the same as at 40 mm particle size, between 75% and 85%. When the particle size increases to 120 mm, the mid line decomposition rate remains around 20% and the decomposition effect is poor, while the near wall line can only reach 50% decomposition rate in the position between the preheating and calcination zones, and in other positions, the decomposition rate is generally below 30%. The main reason of its close its close to 50% decomposition rate is that the limestone inlet is closed when calcination takes place in chamber 1, i.e., a large amount of high temperature exhaust gas accumulates at the top, which promotes limestone decomposition to a certain extent, but this promotion is not stable and the final decomposition effect is far from the decomposition demand.

The undecomposed thickness varies significantly from particle size. The larger the grain size, the more difficult the decomposition and the greater the effect on the final decomposition rate. The limestone decomposition rate is also strongly influenced by the side wall effect, with limestone decomposition in the mid line outperforming that in the near wall line. At the same time, the side wall effect has a greater influence on the small particle size than on the large particle size. At 120 mm particle size, the effect of not reaching the decomposition temperature at the core is much greater than the effect of the side wall effect on decomposition.

## 5. Conclusions

This paper presents a comparative analysis of the gas velocity field, gas temperature field, solid surface and core temperature field, and limestone decomposition field for limestone decomposition processes at 40 mm, 80 mm and 120 mm particle sizes. The results show that.

(i) Changing the fuel gas from natural gas to BFG while keeping the PFR lime kiln structure unchanged will reduce the range of supported calcined grain sizes from 40 mm to 120 mm to below 80 mm, and that limestone of 120 mm grain size is difficult to calcine and decompose under current conditions.
(ii) Even at 40 mm grain size, the decomposition rate is over 90% after calcination by natural gas, whereas with BFG calcination, the decomposition rate is 75% to 85% and the under burning rate is even higher.
(iii) By switching to calcination by BFG, the problem of hard burnt is largely avoided as the solid surface temperature does not exceed 1200 K.
(iv) Analysis of the exhaust gas outlet temperature shows that the exhaust gas is discharged at a temperature of no more than 700 K and even less than 500 K at 40 mm particle size, which is a further improvement in the utilisation of energy.
(v) The side wall effect has a greater impact on the calcination of small grain size limestone and is the main reason for the higher under burning rate.
(vi) There is room for further research and exploration in this study as follows. Firstly, it is clear that the original kiln structure is no longer suitable for BFG calcination of limestone and needs to be optimised for low calorific value fuel calcination of limestone; secondly, the particle size range for BFG calcination of limestone must be less than the range of 40 mm to 120 mm, which is suitable for natural gas calcination, but the exact particle size support range still needs to be further investigated in conjunction with experiments after optimising the PFR lime kiln structure. Finally, experimental validation of low calorific value gas-fired limestone calcination needs to be established and this part of the work will be necessary.

## Figures and Tables

**Table 1:** Calculation conditions.

Calculation Conditions	Value	Unit
Fuel gas inlet velocity	20	m/s
Fuel gas inlet temperature	1673	K
Fuel gas nozzle diameter (8)	70	mm
Cooling air inlet velocity	10	m/s
Cooling air inlet temperature	300	K
Cooling air inlet diameter	1000	mm
Material movement speed	1.54	m/h
Initial temperature of material (limestone)	300	K
Average diameter of material	40, 80, 120	mm
Calcium carbonate density	3310	kg/m^3^
Calcium oxide density	2810	kg/m^3^
Calcium carbonate thermal conductivity	2.26	W/m·k
Calcium oxide thermal conductivity	0.07	W/m·k
Calcium carbonate decomposition temperature	1073	K
Void fraction	0.36, 0.41, 0.46	-

## Nomenclature

$\rho_{g}$ Fluid density; $D_{p}$ Diameter of the particles; *α* Permeability in porous media; *C*_2_ Inertial drag coefficient; $M_{{\mathrm{CaCO}}_{3}}$ Relative molecular mass of calcium carbonate; $d_{{\mathrm{CaCO}}_{3}}$ Diameter of the internal core calcium carbonate; $\rho_{{\mathrm{CaCO}}_{3}}$ density of the calcium carbonate; $R_{D}$ Limestone decomposition rate; $k_{D}$ Reaction constants; $p_{eq}$ Equilibrium partial pressure of carbon dioxide at the front of the reaction zone; $p_{co_{2}}$ CO_2_ partial pressure in the environment; $T_{p}$ Average particle temperature; $T_{i}$ Temperature of internal core calcium carbonate; $Y_{T.C}$ Reaction rate correction factor; $\lambda_{1}$ Thermal conductivity of internal core calcium carbonate, $\lambda_{2}$ Thermal conductivity of external core calcium oxide, $r_{c1}$ Calcium carbonate radius $r_{c1.m}$ 1/2 calcium carbonate radius $r_{c2.m}$ calcium carbonate radius plus 1/2 calcium oxide layer radius; $\varphi_{{\mathrm{CaCO}}_{3}}$ Volume fraction of calcium carbonate, $\rho_{i}$ density of calcium carbonate, $c_{i}$ specific heat capacity of calcium carbonate; $Q_{D}$ Particle reaction rate; $\Delta H_{R}$ Heat of decomposition of calcium carbonate(183,000 J/mol); $V_{p}$ Volume of individual particle; $\varphi_{\mathrm{CaO}}$ Volume fraction of calcium oxide, $\rho_{o}$ density of calcium oxide, $c_{o}$ specific heat capacity of calcium oxide; $a_{v}$ Specific surface area of limestone; $h_{v}$ heat transfer coefficient of gas solid two phase flow; σ Stephen Boltzmanns constant; $5{.6697\;\times \;10}^{-8}{\;W/(K}^{4}\cdot m^{2})$; β Radiation attenuation coefficient of porous media; Nu Nussel number, $l_{z}$ Stacked bed feature length, Pr Prandtl number, Re Reynolds number, a Thermal diffusion coefficient; ν Kinematic viscosity of gas, μ dynamic viscosity of gas, u Velocity of gas movement.
